# Supplementary material for: Comparative genomics provides new insights into the diversity, physiology, and sexuality of the only industrially exploited tremellomycete: Phaffia rhodozyma
Source: BMC Genomics. 2016 Nov 9;17:901. doi: 10.1186/s12864-016-3244-7 (PMC5103461; doi:10.1186/s12864-016-3244-7)
Supplement: Additional file 6: — List of orphan genes with links to PFAM (related to Additional file 1: Table S1). (ZIP 1428 kb) [file 12864_2016_3244_MOESM6_ESM.zip › BLAST_HTML_FTR/G03815_P.html]

BLAST Search Results


```
BLASTP 2.2.27+


Reference:
Stephen F. Altschul, Thomas L. Madden, Alejandro A. Schäffer,
Jinghui Zhang, Zheng Zhang, Webb Miller, and David J. Lipman (1997),
"Gapped BLAST and PSI-BLAST: a new generation of protein database
search programs", Nucleic Acids Res. 25:3389-3402.


Reference for
composition-based statistics:
Alejandro A. Schäffer, L. Aravind, Thomas L. Madden, Sergei
Shavirin, John L. Spouge, Yuri I. Wolf, Eugene V. Koonin, and
Stephen F. Altschul (2001), "Improving the accuracy of PSI-BLAST
protein database searches with composition-based statistics and
other refinements", Nucleic Acids Res. 29:2994-3005.


Database: nr
           71,551,133 sequences; 26,053,659,533 total letters


Query= G03815_P

Length=528
                                                                      Score     E
Sequences producing significant alignments:                          (Bits)  Value

emb|CED83698.1|  hypothetical protein [Xanthophyllomyces dendrorh...   934    0.0  
ref|XP_007368089.1|  hypothetical protein DICSQDRAFT_28489, parti...  42.0    0.20 
gb|EPS94526.1|  hypothetical protein FOMPIDRAFT_139014 [Fomitopsi...  42.0    1.6  
ref|XP_008039413.1|  hypothetical protein TRAVEDRAFT_49002 [Trame...  42.0    1.9  


 >emb|CED83698.1| hypothetical protein [Xanthophyllomyces dendrorhous]
Length=473

 Score =  934 bits (2415),  Expect = 0.0, Method: Compositional matrix adjust.
 Identities = 467/468 (99%), Positives = 468/468 (100%), Gaps = 0/468 (0%)

Query  1    MDEASQIQHFSSKRSSLPSFSSREIPSSPSVSVRRQSFQTQPSEAPLEKKKDGHSGLKRT  60
            MDEASQIQHFSSKRSSLPSFSSREIPSSPSVSVRRQSFQTQPSEAPLEKKKDGHSGLKRT
Sbjct  1    MDEASQIQHFSSKRSSLPSFSSREIPSSPSVSVRRQSFQTQPSEAPLEKKKDGHSGLKRT  60

Query  61   WSMDSVKSDSEESTHPIGTPKREGFQMSNNAHPYQIVSSSSAVLSRGNPTPTKKSWVPTH  120
            WSMDSVKSDSEESTHPIGTPKREGFQMSNNAHPYQIVSSSSAVLSRGNPTPTKKSWVPTH
Sbjct  61   WSMDSVKSDSEESTHPIGTPKREGFQMSNNAHPYQIVSSSSAVLSRGNPTPTKKSWVPTH  120

Query  121  RSSKSLSSMPSDPSYRPRPLPTPPHATLSPSSTTGVHPQLSPCPSDQVEKRGVRNLARKW  180
            RSSKSLSSMPSDPSYRPRPLPTPPHATLSPSSTTGVHPQLSPCPSDQVEKRGVRNLARKW
Sbjct  121  RSSKSLSSMPSDPSYRPRPLPTPPHATLSPSSTTGVHPQLSPCPSDQVEKRGVRNLARKW  180

Query  181  EERGSNSSEASSVEQSETEETRLSDHSQSNREPRSLNEHIVPPNQPNRIFDLNGSPTSEN  240
            EERGSNSSEASSVEQSETEETR+SDHSQSNREPRSLNEHIVPPNQPNRIFDLNGSPTSEN
Sbjct  181  EERGSNSSEASSVEQSETEETRVSDHSQSNREPRSLNEHIVPPNQPNRIFDLNGSPTSEN  240

Query  241  EAYSLLPPSPPVSISSSSDFGSPPLVPTPPALINYSQPSKYSTLSRSTNRTRPKRPSESF  300
            EAYSLLPPSPPVSISSSSDFGSPPLVPTPPALINYSQPSKYSTLSRSTNRTRPKRPSESF
Sbjct  241  EAYSLLPPSPPVSISSSSDFGSPPLVPTPPALINYSQPSKYSTLSRSTNRTRPKRPSESF  300

Query  301  VDRPCADSDGDSDEEDSRSRTFRRNVTARPSKILVTSHITGSIESKKDEADWMVQHSNAP  360
            VDRPCADSDGDSDEEDSRSRTFRRNVTARPSKILVTSHITGSIESKKDEADWMVQHSNAP
Sbjct  301  VDRPCADSDGDSDEEDSRSRTFRRNVTARPSKILVTSHITGSIESKKDEADWMVQHSNAP  360

Query  361  GRETEYRYIIQSLTARIEALEVKLFDLERKSMVPLGSLDSELPPFSAAGHVVSSIPGSST  420
            GRETEYRYIIQSLTARIEALEVKLFDLERKSMVPLGSLDSELPPFSAAGHVVSSIPGSST
Sbjct  361  GRETEYRYIIQSLTARIEALEVKLFDLERKSMVPLGSLDSELPPFSAAGHVVSSIPGSST  420

Query  421  PSRAASSTRERSDGKRNRSVLAGSVPGYLFLVSIGVGIVTWEVLWRRF  468
            PSRAASSTRERSDGKRNRSVLAGSVPGYLFLVSIGVGIVTWEVLWRRF
Sbjct  421  PSRAASSTRERSDGKRNRSVLAGSVPGYLFLVSIGVGIVTWEVLWRRF  468


>ref|XP_007368089.1| hypothetical protein DICSQDRAFT_28489, partial [Dichomitus squalens 
LYAD-421 SS1]
 gb|EJF59144.1| hypothetical protein DICSQDRAFT_28489, partial [Dichomitus squalens 
LYAD-421 SS1]
Length=88

 Score = 42.0 bits (97),  Expect = 0.20, Method: Compositional matrix adjust.
 Identities = 28/85 (33%), Positives = 43/85 (51%), Gaps = 18/85 (21%)

Query  78   GTPKREGFQMSNNAHPYQIVSSSSAVLSRG-----NPTPTKKSWVPT------------H  120
             +P ++   +  N HPY I ++S+ +L+R      N   T   ++PT            H
Sbjct  1    ASPSKKDSALKPNPHPYAIKTTSTGILTRSNSSGYNANATYHHYIPTSPSHNRFDSGKGH  60

Query  121  RSSKSLSSMPSDPSYR-PRPLPTPP  144
            RS+KSL+++P  P    PRPLP PP
Sbjct  61   RSTKSLTAVPESPGREAPRPLPIPP  85


>gb|EPS94526.1| hypothetical protein FOMPIDRAFT_139014 [Fomitopsis pinicola FP-58527 
SS1]
Length=515

 Score = 42.0 bits (97),  Expect = 1.6, Method: Compositional matrix adjust.
 Identities = 30/86 (35%), Positives = 46/86 (53%), Gaps = 20/86 (23%)

Query  90   NAHPYQIVSSSSAVLSRGNPT-----PTKKSWVPT-------------HRSSKSLSSMPS  131
            N HPY + +++SA+L+R N +      T+  +VP              H+ SKSL+++  
Sbjct  35   NPHPYAVKTTTSALLTRSNSSNHNVEATRHYYVPLSPNASRSPESTKGHKISKSLTAVAE  94

Query  132  DPSYRPRPLPTPPHATLSPSSTTGVH  157
             PS  PRPLP PP    S ++T+ VH
Sbjct  95   SPSRSPRPLPAPPMG--SSANTSPVH  118


>ref|XP_008039413.1| hypothetical protein TRAVEDRAFT_49002 [Trametes versicolor FP-101664 
SS1]
 gb|EIW57983.1| hypothetical protein TRAVEDRAFT_49002 [Trametes versicolor FP-101664 
SS1]
Length=775

 Score = 42.0 bits (97),  Expect = 1.9, Method: Compositional matrix adjust.
 Identities = 28/72 (39%), Positives = 38/72 (53%), Gaps = 17/72 (24%)

Query  90   NAHPYQIVSSSSAVLSRG----NPTPTKKSWVPT------------HRSSKSLSSMPSDP  133
            N HPY I ++S+A+L+R     N   T   +VP             HR++KSL+SM   P
Sbjct  33   NPHPYAIKTTSTALLTRSTSEYNVNATHHYYVPLSPHSNKSDVGKGHRATKSLTSMAESP  92

Query  134  SYR-PRPLPTPP  144
            +   PRPLP PP
Sbjct  93   TREAPRPLPIPP  104


Lambda      K        H        a         alpha
   0.309    0.124    0.354    0.792     4.96 

Gapped
Lambda      K        H        a         alpha    sigma
   0.267   0.0410    0.140     1.90     42.6     43.6 

Effective search space used: 5539705996020


  Database: nr
    Posted date:  Sep 23, 2015 12:05 AM
  Number of letters in database: 26,053,659,533
  Number of sequences in database:  71,551,133


Matrix: BLOSUM62
Gap Penalties: Existence: 11, Extension: 1
Neighboring words threshold: 11
Window for multiple hits: 40
```
